# Seroprevalence of acute dengue in a Malaysian tertiary medical centre

**DOI:** 10.12669/pjms.322.9320

**Published:** 2016

**Authors:** Chuan Hun Ding, Zetti Zainol Rashid, Md. Mostafizur Rahman, NanFeng Khang, Wan Ngor Low, Nurabrar Hussin, Melissa Iqlima Marzuki, Alyaa Nadhira Jaafar, Nurul Ain’ Nabilla Roslan, Terukumar Chandrasekaran

**Affiliations:** 1 Chuan Hun Ding, Department of Medical Microbiology & Immunology, Universiti Kebangsaan Malaysia Medical Centre, Kuala Lumpur, Malaysia; 2 Zetti Zainol Rashid, Department of Medical Microbiology & Immunology, Universiti Kebangsaan Malaysia Medical Centre, Kuala Lumpur, Malaysia; 3 Md. Mostafizur Rahman, Department of Medical Microbiology & Immunology, Universiti Kebangsaan Malaysia Medical Centre, Kuala Lumpur, Malaysia; 4 NanFeng Khang, Faculty of Medicine, Universiti Kebangsaan Malaysia, Kuala Lumpur, Malaysia; 5 Wan Ngor Low, Faculty of Medicine, Universiti Kebangsaan Malaysia, Kuala Lumpur, Malaysia; 6 Nurabrar Hussin, Faculty of Medicine, Universiti Kebangsaan Malaysia, Kuala Lumpur, Malaysia; 7 Melissa Iqlima Marzuki, Faculty of Medicine, Universiti Kebangsaan Malaysia, Kuala Lumpur, Malaysia; 8 Alyaa Nadhira Jaafar, Faculty of Medicine, Universiti Kebangsaan Malaysia, Kuala Lumpur, Malaysia; 9 Nurul Ain’ Nabilla Roslan, Faculty of Medicine, Universiti Kebangsaan Malaysia, Kuala Lumpur, Malaysia; 10 Terukumar Chandrasekaran, Faculty of Medicine, Universiti Kebangsaan Malaysia, Kuala Lumpur, Malaysia

**Keywords:** Dengue, DENV, IgG, IgM, NS1, Seroprevalence

## Abstract

**Objectives:**

The aims of this study were to determine the seroprevalence of acute dengue in Universiti Kebangsaan Malaysia (UKM) Medical Centre and its correlation with selected haematological and biochemical parameters.

**Methods:**

This cross-sectional study was conducted from January to June 2015. A patient was serologically diagnosed with acute dengue if the dengue virus IgG, IgM or NS-1 antigen was reactive.

**Results:**

Out of 1,774 patients suspected to have acute dengue, 1,153 were serologically diagnosed with the infection, resulting in a seroprevalence of 64.9%. Dengue-positive patients had a lower mean platelet count (89 × 10^9^/L) compared to the dengue-negative patients (171 × 10^9^/L) (*p*<0.0001). The mean total white cell count was also lower in the dengue-positive cases (4.7 × 10^9^/L vs. 7.2 × 10^9^/L; *p*<0.0001). The mean haematocrit was higher in patients with acute dengue (42.5% vs. 40.0%; *p*<0.0001). Likewise, the serum alanine transaminase level was also higher in patients with acute dengue (108 U/L vs. 54 U/L; *p*<0.0001).

**Conclusions:**

Dengue is very prevalent in UKM Medical Centre as most patients suspected to have acute dengue had serological evidence of the infection. The platelet count was the single most likely parameter to be abnormal (i.e. low) in patients with acute dengue.

## INTRODUCTION

Dengue is caused by a mosquito-borne virus of the same name which belongs to the Flavivirus genus of the Flaviviridae family. The dengue virus (DENV) has four distinct serotypes. While infection with one DENV serotype typically provides lifelong immunity against the same serotype, it does not provide lasting immunity against the other serotypes.^1^ The clinical spectrum ranges from subclinical infection to the potentially fatal dengue shock syndrome.^2^

Dengue is not a new disease and it has been a public health menace since the 1950s.^3^ Tropical countries in Asia are highly vulnerable to the infection and dengue is endemic in Malaysia. A total of 43,346 dengue cases were reported in Malaysia in 2013.^4^ This was a 98% increase compared to the number of cases reported in 2012.^4^ Showing no sign of abatement, the number of dengue cases reported in 2014 spiked further to 108,698, which was a 151% increase compared to the preceding year.^5^

The laboratory diagnosis of dengue is achieved by detecting either viral components or host antibodies mounted against the virus. Thus, for patients presenting early (day 1-7 of illness), the detection of viral non-structural protein 1 (NS1) in sera is recommended.^6^ Dengue-specific antibodies of diagnostic importance are immunoglobulins M and G (IgM and IgG). In primary infections, dengue-specific IgM can be detectable as early as day 4 of illness and dengue-specific IgG by the fourteenth day.^6^, ^7^ In secondary infections both IgG and IgM appear as early as day 2 of illness.^7^

Dengue infection is also associated with anomalies in certain haematological and biochemical parameters. Specifically, leucopenia, thrombocytopaenia, haemo-concentration and elevated serum transaminases can be present in acute dengue.^8^ The aims of this study were to determine the seroprevalence of acute dengue in UKM Medical Centre and its correlation with selected haematological and biochemical parameters.

## METHODS

### Study design and population

This cross-sectional study was conducted in UKM Medical Centre from January to June 2015. The study was approved by the Research Ethics Committee of UKM (approval code: FF-2015-120). During the study period, a total of 1,774 patients clinically suspected to have acute dengue were included for analysis. These patients presented with an acute febrile illness together with at least two other complaints (i.e. arthralgia, myalgia, headaches, retro-orbital pain, petechial rashes or haemorrhagic manifestations). Data on acute dengue status, total white cell count, platelet count, haematocrit, and alanine transaminase (ALT) level were retrieved for each patient from the hospital’s laboratory information system.

### Determination of acute dengue status

DENV antibodies were detected in patients’ sera using Panbio Dengue Duo Cassette, which is an immunochromatographic assay kit (Standard Diagnostics, Inc., Korea). The presence of DENV IgM in isolation would indicate a primary infection. Due to the kit’s high IgG cut off (haemagglutination inhibition titre of at least 1:2560), any reactive IgG result (regardless of the IgM status) would still indicate an acute secondary dengue infection and not a past infection. Dengue NS1 antigen were detected in patients’ sera using diaxis™ Dengue NS1 Antigen Test, which is also an immunochromatographic asssay (AxisBio Diagnostics Sdn. Bhd., Malaysia). A patient was considered to have acute dengue if at least one of DENV IgG, DENV IgM or DENV NS1 antigen was reactive.

### Data analysis

The mean white cell count, mean platelet count, mean haematocrit and mean ALT level were calculated for both the acute dengue-positive and acute dengue-negative groups. The means were then analysed using the unpaired student’s *t*-test. A *p*-value of < 0.05 was considered statistically significant.

## RESULTS

**Fig.1:**
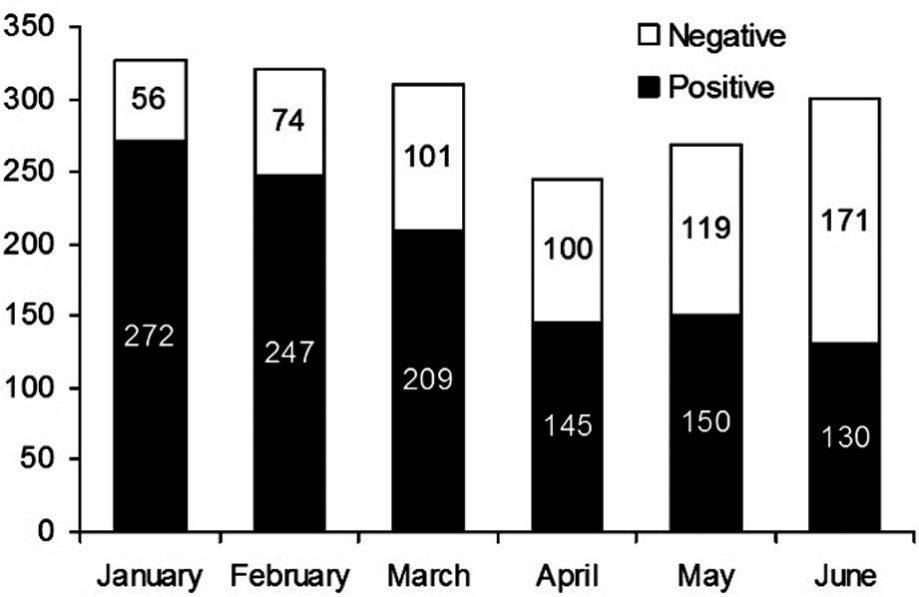
Number of acute dengue-positive and acute dengue-negative cases in the first six months of 2015.

Out of the 1,774 patients clinically suspected to have acute dengue in the study, 1,153 were serologically diagnosed with acute dengue, resulting in a seroprevalence of 64.9%. From January to June of 2015, there has been a general decline in the number of acute dengue cases from 272 in January to 130 cases in June (Fig.1). The male to female ratio for patients with acute dengue was 1.6:1.

Out of the 1,153 patients diagnosed with acute dengue, 516 (44.8%) had serological evidence of primary dengue (positive for DENV IgM in isolation), 471 (40.8%) had secondary dengue (positive for DENV IgG independent of IgM positivity) and 166 (14.4%) patients could not be categorized (NS1 Ag-reactive without DENV antibody test results). Among the haematological and biochemical parameters, only the mean platelet count and mean ALT level were low and elevated in patients with acute dengue, respectively, as presented in Table-I and II. The mean white cell count and haematocrit were within the normal limits in the acute dengue patients. However, when compared to the patients without acute dengue, the means for all four parameters were significantly different between the dengue-positive and dengue-negative groups.

**Table-I:** Haematological parameters in patients with and without acute dengue.

Parameter	Acute dengue-positive patients (n=1134)	Acute dengue-negative patients (n =591)	*p*-value
Total white cell count x 10^9^/L; mean ± SD (range)	4.7 ± 3.9 (1.0 - 87.1)	7.2 ± 5.2 (0.5 - 61.5)	< 0.01
Platelet count x 10^9^/L; mean ± SD (range)	89 ± 69 (5 - 668)	171 ± 100 (10 - 778)	< 0.01
Hematocrit in %; mean ± SD (range)	42.5 ± 5.4 (21.7 – 60.8)	40.0 ± 5.8 (16.2 - 63.5)	< 0.01

**Table-II:** Alanine transaminase level in patients with and without acute dengue.

Parameter	Acute dengue-positive patients (n=1049)	Acute dengue-negative patients (n=467)	*p*-value
Alanine transaminase level in U/L; mean ± SD (range)	108 ± 151 (6 - 2521)	55 ± 63 (6 - 609)	< 0.01

## DISCUSSION

For the past couple of years, Malaysia’s national statistics have been far from optimistic, with an ever increasing number of dengue cases being reported despite the various vector-control programmes by the local health authorities such as fogging, punitive fines for premises with mosquito larvae and dengue awareness campaigns. However, during the first half of this year, there was a decreasing trend at UKM Medical Centre. This could be due to the different geographical origins of the centre’s patients in the different months and the recent inter-ministerial cooperation initiative launched by the government to reduce the number of dengue cases.

Dengue is one of several infectious diseases associated with thrombocytopenia.^6^ However, although it is a non-specific finding, it is often associated with dengue in areas of high dengue endemicity. This study’s significant association between acute dengue and thrombocytopenia parallels those of other investigators.^6^, ^9^ Decreased platelet production from the bone marrow, increased peripheral destruction of platelets or immune-complex lysis are the three main mechanisms of thrombocytopenia in dengue.^9^ However, there were a few dengue-positive patients with thrombocytosis in this study. The possible causes are comorbidities such as myeloproliferative disorders (e.g. polycythaemia vera), various malignancies and hyposplenism.^10^

Leucopenia has been reported to have a good predictive value in acute dengue.^11^ Although this study revealed that the mean white cell count was significantly lower in dengue-positive patients, the leucocyte mean was within the normal range in both dengue-positive and dengue-negative patients. It has been postulated that leucopenia occurs during the febrile period of dengue infection as a consequence of destruction of bone marrow precursor cells by DENV.^12^ One patient with acute dengue actually had leucocytosis (87.10 ×10^9^/L). He was concurrently suffering from acute myeloid leukemia. Bacterial co-infection is another possible explanation for leucocytosis in the setting of an acute dengue infection.^13^

Haemoconcentration is an indicator of plasma leakage in certain forms of dengue (e.g. dengue haemorrhagic fever). However, this study found that the mean haematocrit level was within the normal range in dengue-positive patients although the mean was significantly higher compared to the dengue-negative patients. Possible reasons include a normal haematocrit early in the course of an acute dengue infection,^14^ or the haemoconcentration may be masked by early fluid replacement. Thus, the haematocrit is not reliable as a diagnostic marker for acute dengue infection. Its importance lies mainly in the monitoring of patients with acute dengue for clinical deterioration.^14^

Acute dengue is associated with a mild to moderate elevation in serum ALT levels.^15^ Consistent with what has been reported by other investigators, this study also found that patients with acute dengue tend to have significantly higher mean ALT levels compared to the dengue-negative cases. The raised ALT is attributable to hepatic injury caused by direct infection of hepatocytes and kupffer cells by DENV.^15^ As hepatic involvement is common in DENV infection, dengue fever should be considered as one of the differential diagnoses of acute hepatitis in dengue endemic areas.^16^ Like the other serum parameters studied, raised ALT is also not specific to dengue as some of the dengue-negative cases in this study also had elevated ALT levels, albeit lower than the dengue-positive cases.

### Limitations of the Study

The important limitations of this study were the relatively short study duration of six months and that we did not correlate the parameters with day of illness. This study’s finding with regards to the seroprevalence of acute dengue is applicable only to the geographical area where UKM Medical Centre is located and larger (multi-centre) studies are recommended. We also relied solely on serological test results to label patients with acute dengue without using a second test (e.g. genomic detection) to confirm their virological status.

In conclusion, dengue is very prevalent in UKM Medical Centre as most patients suspected to have acute dengue clinically had serological evidence of the infection. As the management of dengue is supportive, it is important to identify those patients who are likely to have the infection so that they may be admitted for vital signs monitoring and (re)hydration. The platelet count was the single most likely parameter to be abnormal (i.e. low) in these patients. Thus, all patients with thrombocytopaenia and fever in Malaysia should be investigated further with serological tests which detect either NS1 antigen or dengue-specific antibodies.
